# A novel machine learning algorithm, Bayesian networks model, to predict the high‐risk patients with cardiac surgery‐associated acute kidney injury

**DOI:** 10.1002/clc.23377

**Published:** 2020-05-12

**Authors:** Yang Li, Jiarui Xu, Yimei Wang, Yunlu Zhang, Wuhua Jiang, Bo Shen, Xiaoqiang Ding

**Affiliations:** ^1^ Department of Nephrology, Zhongshan Hospital Fudan University Shanghai China; ^2^ Shanghai Medical Center of Kidney Shanghai China; ^3^ Shanghai Key Laboratory of Kidney and Blood Purification Shanghai China; ^4^ Shanghai Institute of Kidney and Dialysis Shanghai China; ^5^ Hemodialysis Quality Control Center of Shanghai Shanghai China

**Keywords:** acute kidney injury, Bayesian networks, cardiac surgery, disease prediction, machine learning

## Abstract

**Background:**

Cardiac surgery‐associated acute kidney injury (CSA‐AKI) is a well‐recognized complication with an ominous outcome.

**Hypothesis:**

Bayesian networks (BNs) not only can reveal the complex interrelationships between predictors and CSA‐AKI, but predict the individual risk of CSA‐AKI occurrence.

**Methods:**

During 2013 and 2015, we recruited 5533 eligible participants who underwent cardiac surgery from a tertiary hospital in eastern China. Data on demographics, clinical and laboratory information were prospectively recorded in the electronic medical system and analyzed by gLASSO‐logistic regression and BNs.

**Results:**

The incidences of CSA‐AKI and severe CSA‐AKI were 37.5% and 11.1%. BNs model revealed that gender, left ventricular ejection fractions (LVEF), serum creatinine (SCr), serum uric acid (SUA), platelet, and aortic cross‐clamp time (ACCT) were found as the parent nodes of CSA‐AKI, while ultrafiltration volume and postoperative central venous pressure (CVP) were connected with CSA‐AKI as children nodes. In the severe CSA‐AKI model, age, proteinuria, and SUA were directly linked to severe AKI; the new nodes of NYHA grade and direct bilirubin created relationships with severe AKI through was related to LVEF, surgery types, and SCr level. The internal AUCs for predicting CSA‐AKI and severe AKI were 0.755 and 0.845, which remained 0.736 and 0.816 in the external validation. Given the known variables, the risk for CSA‐AKI can be inferred at individual levels based on the established BNs model and prior information.

**Conclusion:**

BNs model has a high accuracy, good interpretability, and strong generalizability in predicting CSA‐AKI. It facilitates physicians to identify high‐risk patients and implement protective strategies to improve the prognosis.

## 1 INTRODUCTION

Along with the enhancement of medical technology, considerable progress has been achieved in the surgical treatments of cardiac diseases. Each year, an estimated 2 million cardiac surgeries are performed worldwide.^1^ Acute kidney injury (AKI) is a well‐recognized complication following cardiac surgery. The pooled incidence of cardiac surgery‐associated acute kidney injury was 24.3% from a global meta‐analysis.^2^ Furthermore, severe CSA‐AKI is positively associated with a higher mortality, prolonged length of hospital stay, and increased medical cost.^3^ The occurrence of CSA‐AKI involves both demographic and perioperative factors, and the specific mechanism and severity vary with the individual.^4^

Early identification of high‐risk CSA‐AKI patients, with the application of prediction models, allows clinicians to monitor these patients periodically and take prophylaxis to prevent the occurrence of AKI. Several risk prediction models for AKI have been developed based on logistic regression, such as *Clinic Score*,^5^ *Mehta Score*,^6^ and the *Simplified Renal Index Score*.^7^ Yet, despite the findings that good performance was reported in internal evaluation, most of these models had poor discrimination in the external validation with an area under the receiver‐operator characteristic curves (AUC) below 0.7.^8^, ^9^ Hence, it is necessary to apply advanced algorithms to develop a more flexible and efficient model to identify AKI, especially severe AKI, in an early stage. Bayesian networks (BNs) is considered as one of the classical machine learning algorithms. It can not only graphically exhibit the relationships between casual or associated variables in a network, but also quantitatively define the conditional probability of each node. In the field of nephron‐epidemiology, this characteristic makes BNs more applicable to verify the multiple etiology hypothesis of CSA‐AKI.

To this end, we conduct a prospective cohort in patients who underwent cardiac surgeries. The objective is to propose the BNs‐based predictive model for revealing the complex inherent relations between CSA‐AKI and its associated factors, and then to evaluate the model's predictive ability and external practicality.

## 2 METHODS

### 2.1 Patient selection

From January 1st 2013 to December. 31st 2015, patients receiving cardiac surgeries from a tertiary hospital in Shanghai of China were enrolled as the study participants. We further excluded those who were under 18 years old, receiving the heart transplant, lacked surgery, and biochemical data, or took less than one serum creatinine (SCr) test. Then the eligible participants were subdivided participants into two cohorts. Patients admitted in 2013 to 2014 were assigned as a derivation cohort, and used for statistical analysis, BNs modeling, and internal evaluation. The rest patients in 2015 were designated as a validation cohort to verify the model's external generalizability. The study has been approved by the Zhongshan hospital institutional board (B2017‐039). Participation was voluntary and anonymous, with the confidentiality of patients' identity information assured. Before data collection, informed consent was signed by all participants or their agents.

### 2.2 Data collection

We applied a self‐designed questionnaire to collect demographic information and preexisting comorbidities. Data on perioperative factors were retrieved along with its time records in the electronic medical records. Laboratory indicators within 24 hours at admission were used as the baseline biochemical levels. We finally selected 27 medical indicators that were common in cardiac surgery. These variables were divided chronologically into four groups: ① demographic features: age, gender, body mass index (BMI); ② preoperative features: hypertension, diabetes, coronary angiography, New York heart association (NYHA) grade, left ventricular ejection fractions (LVEF), alanine aminotransferase (ALT), aspartate aminotransferase (AST) and direct bilirubin (DBil), SCr, estimated glomerular filtration rate (eGFR), serum uric acid (SUA), urine protein, urine erythrocyte, album, hemoglobin, hematocrit, platelet, serum sodium, and potassium; ③ intraoperative features: cardio‐pulmonary bypass (CPB), surgery type, aortic cross‐clamp time (ACCT), and ultrafiltration volume; ④ postoperative feature: central venous pressure (CVP) within 6 hours.

### 2.3 Definition and classification

According to the 2012 KDIGO criteria,^10^ AKI is diagnosed as the absolute value of the SCr increase ≥0.3 mg/dL (≥26.5 μmol/L) within 48 hours or an increase ≥1.5 times baseline levels within 7 days, or a urine output <0.5 mL/kg/h lasting over 6 hours. Severe AKI refers to an SCr increase ≥2.0 times baseline level or ≥ 4.0 mg/dL or initiation of renal replacement therapy (RRT). Cardiac surgery was classified as the valve, coronary artery bypass grafting (CABG), aorta, valve+CABG, valve+large vessels, and others. The reference range of eGFR and SUA was ≥90 mL/min/1.73m^2^ and ≤ 359 μmol/L, respectively. The reference range of serum sodium and potassium was 137 ~ 147 mmol/L and 3.5 ~ 5.3 mmol/L, respectively. Values out of the reference range were considered as hypo‐electrolytemia or hyper‐electrolytemia.

### 2.4 gLASSO‐logistic regression

Multicollinearity and high dimensionality are often encountered in clinical data. If not handled properly, they can lead to incorrect parameter estimates or wrong inferences. The LASSO (Least absolute shrinkage and selection operator) method is a shrinkage estimation method proposed by *Tibshirani* (1996).^11^ LASSO can apply a penalty term *l*_*1*_, which is based on the ordinary least squares estimation, and compress the regression coefficients *β* of irrelevant variables to zero, thereby achieving model estimation and variable selection. The gLASSO (group LASSO) is an extension of LASSO, which can select the whole categorical variable as predefined, instead of the single dummy variables.^12^ The expression of gLASSO is

$${\hat{\beta }}_{\text{gLASSO}}=\text{argmin}\left({\left\|Y-\mathrm{X\beta}\right\|}_{2}^{2}+\sum_{g=1}^{G}\lambda_{g}{\left\|\beta_{I_{g}}\right\|}_{2}\right)$$

where *G* is the number of groups and *I*_*g*_ is the variable set of *g*^*th*^ group, *g* = 1, 2, …, *G*. *λ*_*g*_ refers to the penalty parameter of *g*^*th*^ group, which can be regarded as an intermediate between the *l*_*1*_ *‐* and *l*_*2*_ *‐* type penalty.

### 2.5 Bayesian networks

Bayesian networks was first proposed by Pearl Judea in 1988 and widely used in the field of machine learning.^13^ It contains a directed acyclic graph (DAG) *G = (V*, *A)* and a global probability distribution. In DAG, each node *v*_*i*_ ∈ *V* corresponds to a random variable *X*_*i*_. The global probability distribution can be decomposed into smaller conditional probability distributions (CPD) according to the edges *a*_*ij*_ ∈ *A* in the DAG. The factorization of BN' global distribution is specified as:

$$P\left(X_{1},\ldots ,X_{n}\right)=P\left(X_{1}\right)P\left(X_{2}|X_{1}\right)\ldots P\left(X_{n}|X_{1},X_{2},\ldots ,X_{n-1}\right)=\prod_{i=1}^{n}P\left(X_{i}|\pi \left(X_{i}\right);\theta \left(X_{i}\right)\right)$$

where *π(X*_*i*_ *)* is a set of parent variables of *Xi*. Given the value of *π(X*_*i*_ *)*, each node *X*_*i*_ is conditionally independent of its non‐descendants. Building a BNs model requires two steps: structure learning and parameter learning. Tabu‐search is one of the advanced algorithms in structure learning. Compared with K2 and hill‐climbing, Tabu‐search can escape local optima, by random restarts as well as single‐arc addition, removal and reversals, to achieve an optimal network with minimized score function.^14^ Parameter learning refers to defining the numerical parameters of each local distribution by using either maximum likelihood (ML) estimation or Bayesian estimation. ML estimation aims at finding the value of parameter *θ*, which maximizes the likelihood *P*(*X*_*i*_| *θ*), and it is written as $\tilde{\theta }={\text{argmax}}_{\theta }P\left(X_{i}|\theta \right)$. BNs inference boils down to finding a posterior distribution by applying the Bayesian rules. If we call E as the set of observed variables (Evidence), and Z as the set of target or non‐observed variables, then computing an inference on a graphical model finds:

$$P\left(Z|E,\theta \right)=\frac{P\left(Z,E|\theta \right)}{P\left(E|\theta \right)}=\frac{P\left(Z,E|\theta \right)}{\sum_{z\in Z}P\left(Z=z,E|\theta \right)}$$

### 2.6 Statistical analysis

The distributional differences of covariates between the derivation and validation cohorts were assessed by using the standardized differences (SD). SD can distinguish the clinical difference, rather than statistical significance, in a large sample size. If the SD values of most variables exceeded the threshold of 10%, participants in two cohorts were considered to originate from different source populations. In the derivation cohort, we further described the distribution of CSA‐AKI and severe AKI in different clinical factors. Then we quantified their association strength with the adjusted odds ratios (aOR) by using multiple logistic regression. The analysis was conducted in IBM SPSS 22.0 (IBM Corp., Armonk, New York) with a significance level of 0.05. The gLASSO‐logistic regression was run in “grpreg” packages of R program 3.6.0 (R core team) to select predictors of CSA‐AKI from candidate variables. In *gLasso* penalty algorithm, the 10‐fold cross‐validation was applied to plot the set of the regularization parameter λ. When cross‐validation error reached the minimized value (log[λ.min]), the optimal variables were selected. After that, these predictors were used for BNs modeling in “bnlearn” packages of R program. The structure of BNs was learned under the tabu‐search algorithm, while the ML estimation was chosen to acquire the CPD parameters. Model visualization was done in Netica 5.18 (Norsys Software Corp., Vancouver, BC, Canada). Lastly, the predictive ability of BNs models was validated, in both the derivation and validation cohorts, by using precision rate, recall rate, F‐measure value, and AUCs. The related analysis was run in Weka 3.8.0 software (Waikato Environment for Knowledge Analysis, the University of Waikato, New Zealand).

## 3 RESULTS

### 3.1 Baseline characteristics and CSA‐AKI incidence

In total, 5533 patients were enrolled in the final analysis. Of them, 3639 patients were assigned to the derivation cohort and 1894 patients to the validation cohort (Supplementary Figure S1). In the derivation cohort, the average age was 55.0 ± 13.2 years, and 59.6% was male, while the average age was 55.8 ± 13.0 years, and 57.5% was male in the validation cohort. Most of the covariates shared a < 10% SD value, indicating that participants from two cohorts were clinically comparable (Supplementary Table S1).

### 3.2 Preoperative risk factors associated with CSA‐AKI

**FIGURE 1:**
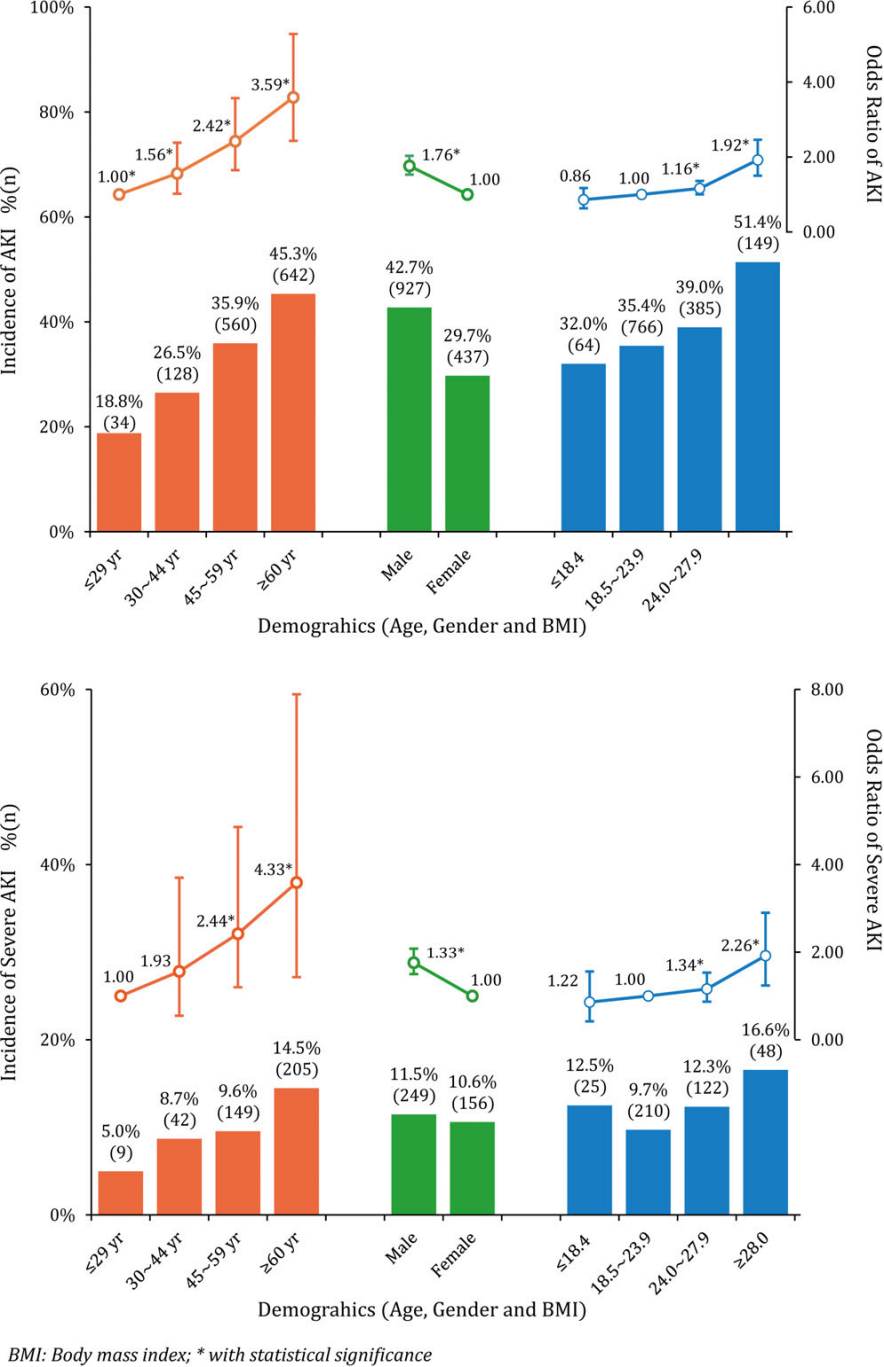
CSA‐AKI and severe AKI incidence in varied demographics among patients receiving cardiac surgery

In the derivation cohort, 1364 patients were diagnosed with CSA‐AKI (37.5%). Of them, 405 patients developed to severe AKI quickly. In Figure 1, male patients shared a relatively higher AKI risk (42.7% vs 29.7%). Dividing age into four levels, the incidence of CSA‐AKI increased significantly from 18.8% in the youngest group to 45.3% in patients over 60 years of age. Obesity (BMI≥28) also increased the incidence of AKI (51.4%). Table 1 showed that poor cardiac conditions (NYHA grade ≥ 3, LVEF<50%) were associated with a substantially higher risk of CSA‐AKI. Patients with a higher DBiL, eGFR, and SUA levels before surgery also increase the risks of developing CSA‐AKI (aOR ranged from 1.20 to 2.63). Coronary angiography was not found a significant association with both AKI and severe AKI. When patients had proteinuria and hematuria at admission, the aOR of CSA‐AKI increased to 2.24 and 1.30. Other biochemical risk factors included anemia, hypoalbuminemia, thrombocytopenia, and so forth. Besides, the above‐mentioned features were identified as the risk factors for severe AKI but shared higher aOR values. One exception was that hyponatremia and hyperkalemia were more positively associated with severe AKI rather than AKI.

**TABLE 1:** Perioperative factors of CSA‐AKI and severe AKI in patients with cardiac surgery in the derivation cohort (n = 3639)

Variables	Total	CSA‐AKI		Severe CSA‐AKI	
		n (%)	aOR^a^ (95% CI)	n (%)	aOR^a^ (95% CI)
*Comorbidities*					
Hypertension	1142	504(44.1)	1.13(0.97 ~ 1.31)	156(13.7)	1.22(0.96 ~ 1.55)
Diabetes	366	147(40.2)	0.85(0.67 ~ 1.06)	43(11.7)	0.83(0.58 ~ 1.18)
*Coronary angiography*					
No	2004	700(34.9)	1.00	210(10.5)	1.00
Yes (within 3 days)	408	162(39.7)	0.86(0.68 ~ 1.08)	52(12.7)	0.92(0.65 ~ 1.31)
Yes (outside 3 days)	1227	502(40.9)	0.99(0.84 ~ 1.16)	143(11.7)	0.92(0.72 ~ 1.18)
*NYHA grade*					
1–2	1531	503(32.9)	1.00	131(8.6)	1.00
3–4	2108	861(40.8)	1.38(1.20 ~ 1.59)	274(13.0)	1.67(1.33 ~ 2.10)
*LVEF*					
≥50%	3209	1153(35.9)	1.00	331(10.3)	1.00
<50%	430	211(49.1)	1.52(1.24 ~ 1.87)	74(17.2)	1.94(1.44 ~ 2.61)
*Liver function*					
ALT (≥40 U/L)	484	183(37.8)	0.95(0.77 ~ 1.16)	53(11.0)	0.98(0.71 ~ 1.35)
AST (≥35 U/L)	496	209(42.1)	1.25(1.03 ~ 1.52)	72(14.5)	1.53(1.14 ~ 2.04)
DBiL (≥20.4 μmol/L)	507	223(44.0)	1.50(1.23 ~ 1.83)	92(18.1)	2.43(1.84 ~ 3.19)
*Renal function*					
SCr (<115 μmol/L)	3431	1226(35.7)	1.00	351(10.2)	1.00
SCr (≥115 μmol/L)	208	138(66.3)	1.67(1.43 ~ 1.94)	54(26.0)	2.05(1.69 ~ 2.49)
eGFR (≥90 mL/min/1.73m^2^)	1722	548(31.8)	1.00	162(9.4)	1.00
eGFR (60 ~ 89 mL/min/1.73m^2^)	1624	640(39.4)	1.20(1.04 ~ 1.40)	177(10.9)	1.11(0.87 ~ 1.41)
eGFR (≤59 mL/min/1.73m^2^)	293	176(60.1)	2.63(2.02 ~ 3.42)	66(22.5)	3.19(2.23 ~ 4.56)
SUA (≤359 μmol/L)	1942	590(30.4)	1.00	154(7.9)	1.00
SUA (360 ~ 419 μmol/L)	790	314(39.7)	1.34(1.12 ~ 1.61)	102(12.9)	1.83(1.39 ~ 2.42)
SUA (≥420 μmol/L)	907	460(50.7)	2.06(1.74 ~ 2.44)	149(16.4)	2.79(2.15 ~ 3.62)
Urine Protein	168	93(55.4)	2.24(1.62 ~ 3.08)	43(25.6)	3.66(2.42 ~ 5.52)
Urine Erythrocyte	456	176(38.6)	1.30(1.05 ~ 1.60)	72(15.8)	1.73(1.28 ~ 2.34)
*Biochemical test*					
Album (<35 g/L)	172	81(47.1)	1.47(1.08 ~ 2.02)	25(14.5)	1.60(1.01 ~ 2.55)
Hemoglobin (<115 g/L)	400	158(39.5)	1.30(1.04 ~ 1.63)	55(13.8)	1.44(1.04 ~ 2.00)
Hematocrit (<30%)	53	28(52.8)	2.17(1.25 ~ 3.80)	5(9.4)	1.37(0.51 ~ 3.66)
Platelet (≤125*10^9^)	406	200(49.3)	1.69(1.37 ~ 2.09)	67(16.5)	1.94(1.43 ~ 2.64)
*Electrolyte disorders*					
Hyponatremia	62	28(45.2)	1.35(0.81 ~ 2.27)	13(21.0)	2.28(1.18 ~ 4.41)
Hypernatremia	124	44(35.5)	0.83(0.57 ~ 1.21)	13(10.5)	0.84(0.46 ~ 1.54)
Hypokalemia	147	66(44.9)	1.42(1.01 ~ 2.00)	23(15.6)	1.67(1.03 ~ 2.71)
Hyperkalemia	30	15(50.0)	1.85(0.89 ~ 3.88)	7(23.3)	3.15(1.25 ~ 7.97)
*CPB*					
Yes	2619	1020(38.9)	1.97(1.67 ~ 2.33)	327(12.5)	2.60(1.97 ~ 3.44)
No	1020	329(32.3)	1.00	78(7.6)	1.00
*Surgery type*					
Valve	1935	689(35.6)	1.72(1.42 ~ 2.09)	191(9.9)	2.42(1.71 ~ 3.43)
CABG	756	254(33.6)	1.00	51(6.7)	1.00
Aorta	108	72(66.7)	6.54(4.17 ~ 10.25)	38(35.2)	19.43(10.9 ~ 34.64)
Valve + CABG	167	104(62.3)	3.58(2.52 ~ 5.10)	40(24.0)	6.85(4.15 ~ 11.29)
Valve + large vessels	235	130(55.3)	3.45(2.52 ~ 4.72)	41(17.4)	5.65(3.49 ~ 9.15)
Others	438	115(26.3)	1.50(1.12 ~ 2.01)	44(10.0)	2.99(1.87 ~ 4.77)
*ACCT*					
No	1020	329(32.3)	1.00	78(7.6)	1.00
≤59 min	1507	468(31.1)	1.36(1.13 ~ 1.63)	125(8.3)	1.51(1.10 ~ 2.07)
60 ~ 119 min	1037	517(49.9)	3.02(2.49 ~ 3.67)	177(17.1)	4.35(3.19 ~ 5.92)
≥120 min	75	50(66.7)	7.26(4.29 ~ 12.29)	25(33.3)	15.42(8.12 ~ 29.30)
*Ultrafiltration volume*					
≤1999 mL	1086	337(31.0)	1.00	81(7.5)	1.00
2000 ~ 2999 mL	2214	850(38.4)	1.37(1.17 ~ 1.61)	258(11.7)	1.71(1.31 ~ 2.24)
≥3000 mL	339	177(52.2)	2.36(1.83 ~ 3.05)	66(19.5)	3.63(2.50 ~ 5.27)
*Postoperative CVP*					
≤7 mmHg	1340	260(19.4)	1.00	49(3.7)	1.00
8 ~ 9 mmHg	867	354(40.8)	2.88(2.37 ~ 3.50)	100(11.5)	4.41(3.07 ~ 6.33)
≥10 mmHg	1432	750(52.4)	4.53(3.80 ~ 5.39)	256(17.9)	8.42(6.09 ~ 11.64)

*Abbreviations*: ACCT, aortic cross‐clamp time; ALT, alanine aminotransferase; aOR, adjusted odds ratio; AST, aspartate aminotransferase; CABG, coronary artery bypass grafting; CI, confidence interval; CPB, cardiac pulmonary bypass; CSA‐AKI, cardiac surgery associated acute kidney injury; CVP, central venous pressure; DBil, direct bilirubin; eGFR, estimated glomerular filtration rate; LVEF, left ventricular ejection fractions; NYHA, New York heart association; SCr, serum creatinine; SUA, serum uric acid.

a OR was adjusted by age, gender, and body mass index.

### 3.3 Intraoperative and postoperative risk factors associated with CSA‐AKI

Table 1 also demonstrates that patients who underwent complicated cardiac surgery, such as the aorta, valve+CABG or larger vessels, were more susceptible to CSA‐AKI (aOR ranged from 3.45 to 6.54) and severe AKI (aOR ranged from 5.65 to 19.43). The utilization of CPB, ACCT, and ultrafiltration volume also has marked impacts on developing CSA‐AKI. With the increase of CVP level after surgery, the risk of AKI also kept growing. Patients with a CVP of ≥10 mmHg had an adjusted odds ratio of 4.53 for AKI and 8.42 for severe AKI.

### 3.4 Variable selection in gLASSO‐logistic regression

Before BNs modeling, we applied gLASSO‐logistic regression to perform variable selections for both CSA‐AKI and severe AKI. When log(λ.min) reached‐4.63, twelve predictors of CSA‐AKI were selected from the original feature‐pool (Supplementary Figure S2). It included age, gender, BMI, SCr, SUA, proteinuria, LVEF, platelet, surgery type, ACCT, ultrafiltration volume, and postoperative CVP level. Following the same steps with a log(λ.min) of −4.69, thirteen not identical variables were selected for severe CSA‐AKI prediction, including age, BMI, SCr, SUA, NYHA grade, LVEF, platelet, DBiL, proteinuria, surgery type, ACCT, ultrafiltration volume, and CVP level. Then, we presented these predictors into multiple logistic regression to quantify the association strength with CSA‐AKI and severe AKI (Table 2).

**TABLE 2:** CSA‐AKI and severe AKI predictors selected by gLASSO‐Logistic regression

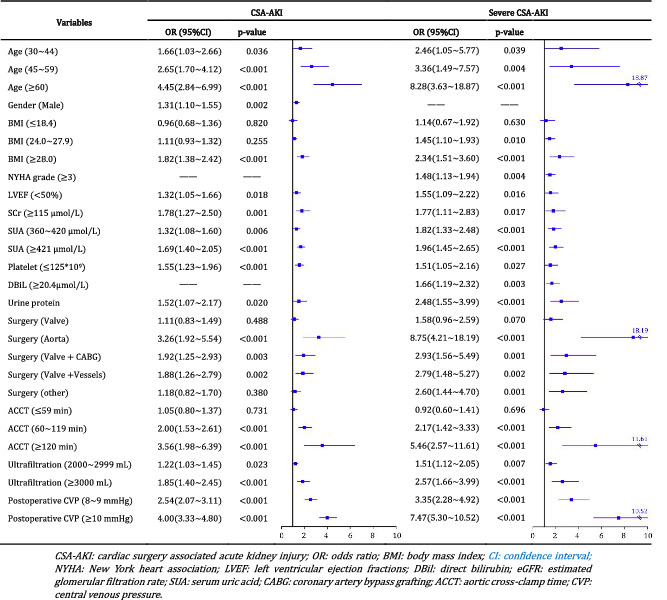

### 3.5 BNs establishment and model inference

**FIGURE 2:**
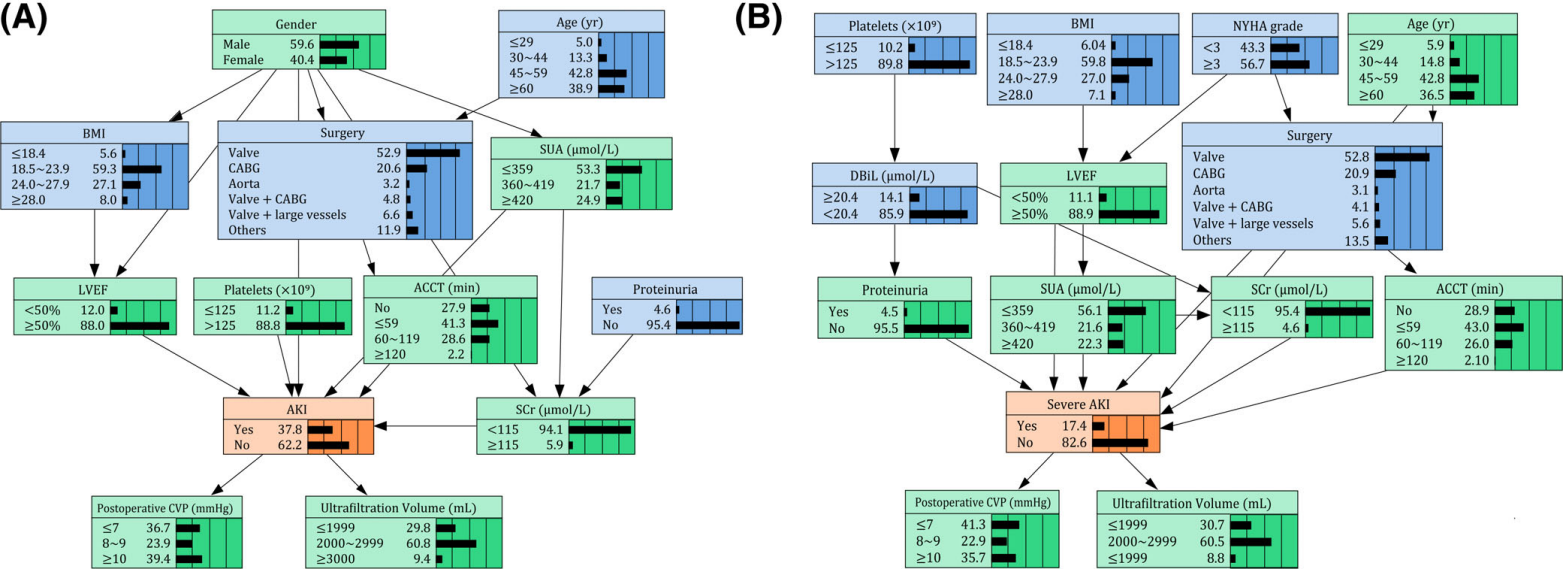
Bayesian networks model of risk factors of CSA‐AKI and severe AKI

The predictive models for CSA‐AKI and severe CSA‐AKI were constructed separately by using BNs analysis. Each predictor was represented by a node, and its relationships with other nodes were linked through an edge. In the CSA‐AKI model, there were 13 nodes and 18 directed edges between CSA‐AKI and its predictors. Figure 2A shows the complex network structure of CSA‐AKI model, in which gender, LVEF, SCr, SUA, platelet, and ACCT were observed as the parent nodes of AKI. Mechanical ventilation and postoperative CVP were also connected with CSA‐AKI as children nodes. Surgery type was indirectly linked to CSA‐AKI through ACCT, while proteinuria created connections with AKI through affecting SCr level. Meanwhile, the severe CSA‐AKI model enrolled 14 nodes and 19 edges, where most of the variates and relationships were similar to those in the CSA‐AKI model (Figure 2B). The specific‐changed relationships in the severe CSA‐AKI model were as follows: age and proteinuria were directly linked to severe AKI; the new node of NYHA grade was related to LVEF and surgery type, which created a relationship with severe AKI through SUA and ACCT; DBiL had indirect connections with severe AKI through proteinuria and SCr level.

Given the known evidence variables, we can infer the risk for developing CSA‐AKI based on the established BNs model. For example, when a 50‐year‐old male patient received 1.5 hours ACCT and 3500 mL ultrafiltration during valve surgery and maintained a postoperative CVP of 9 mmHg, the maximum posterior probability of AKI was 71.6% based on prior information acquired from the model (Supplementary Figure S3A). While if ACCT and ultrafiltration were adjusted at the minimum level and correcting the CVP timely, the incidence of AKI reduced to 16.1% notably (Supplementary Figure S3B).

### 3.6 Predictive ability of BNs model in the internal, 10‐fold cross, and external validation

**FIGURE 3:**
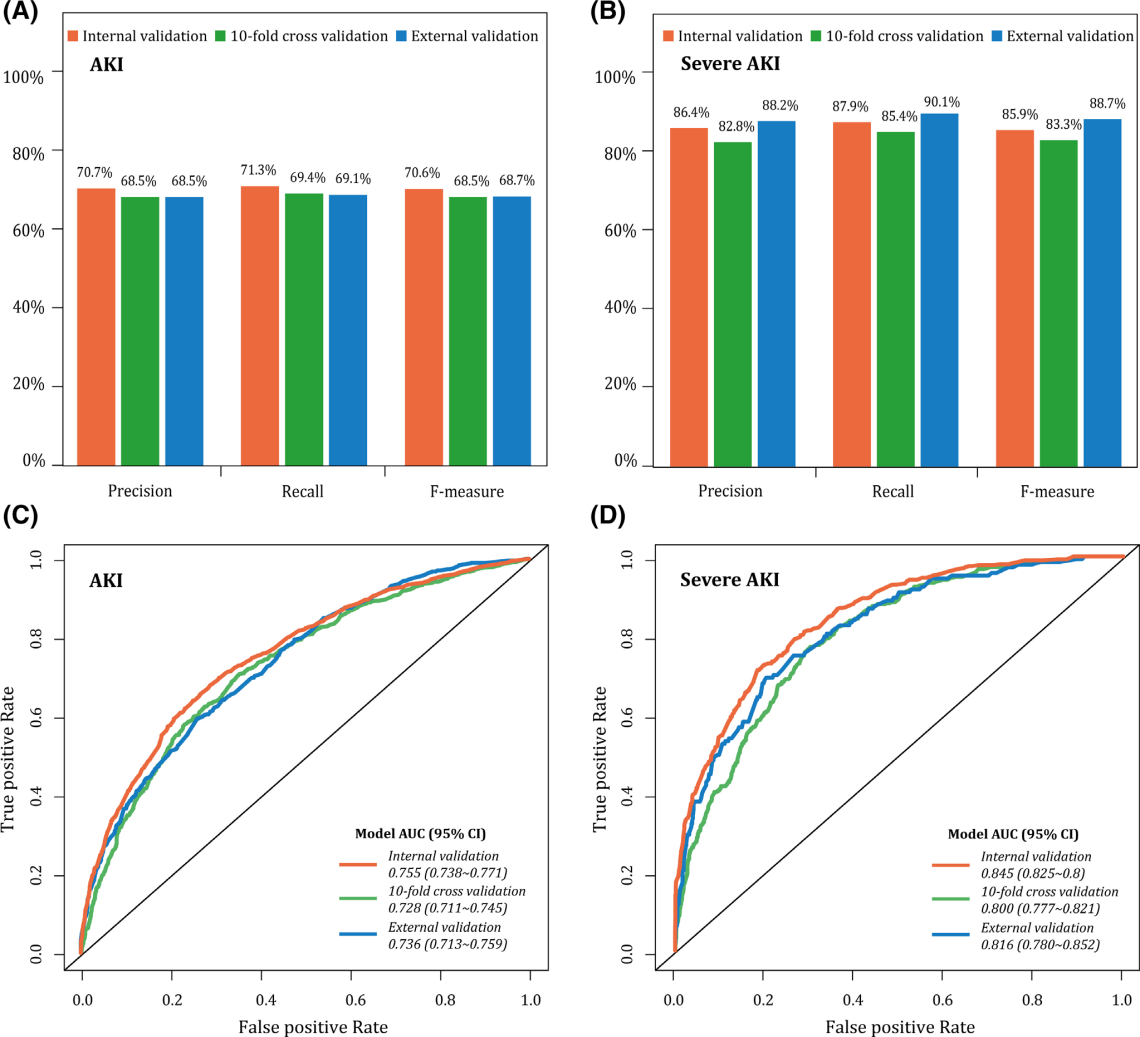
Model evaluation in the internal, 10‐fold cross, and external validation

The precision rate for predicting CSA‐AKI was about 70% in both internal and external validation (Figure 3A). It suggested that BNs model has an advanced suitability for predicting CSA‐AKI. Notably, in the severe AKI model, the F‐measure value was up to 85.9% in internal validation and 88.7% in external validation, demonstrating a good agreement between the actual observations and the BNs predictions for the risk of severe AKI (Figure 3B). Figure 3C,D provides the AUCs for both CSA‐AKI and severe AKI. It was observed that the internal AUCs for predicting CSA‐AKI and severe‐AKI were 0.755 and 0.845. In the process of external validation, AUCs also remained at levels of 0.736 and 0.816. The Mantel‐Haenszel test showed that the differences of predictive accuracy were not statistically significant among internal, 10‐fold cross, and external validation datasets (*P* = .106 and .229 in CSA‐AKI model and severe AKI model, respectively).

## 4 DISCUSSION

In the present study, the incidence of CSA‐AKI was estimated at 37.5%. It is consistent with reported literature, ranging from 3.1% to 42% according to the source population and AKI definition.^15^, ^16^ Among these AKI cases, nearly one third (405/1364) could quickly develop to severe condition (stage 2‐3) within a short time. CSA‐AKI can be described as the type‐1 cardiorenal syndrome, inducing an acute or chronic dysfunction of the heart and kidneys. An abrupt deterioration of cardiac or renal function might trigger pathophysiologic disorder of the other organ.^17^ Apart from nephrologists, physicians in other departments usually ignore the early detection of AKI, due to a lack of dynamic SCr monitoring. One cross‐sectional survey in China reported that over 70 % of hospitalized patients with identifiable AKI could not be recognized.^18^

The occurrence of CSA‐AKI was affected by a range of risk factors. It refers to not only the demographic features such as age, gender, and comorbidity, but also the perioperative factors such as the surgery complexity, ACCT, ultrafiltration volume, and whether or not CPB are given. The main pathways of CSA‐AKI include hypoperfusion, ischaemia‐reperfusion injury, neurohumoral activation, inflammation, oxidative stress, nephrotoxins, and mechanical factors.^4^, ^19^

In this study, we applied BNs to develop predictive models of AKI and severe AKI in patients with cardiac surgery. Through the directed acyclic graph, the complex relationships between risk factors and AKI were delineated intuitively. Importantly, these interdependencies are consistent with the biological and clinical interpretations.^20^, ^21^ For example, patients with complicated cardiac surgeries and prolonged ACCT are more likely to develop into CSA‐AKI. Yet, such high‐order interaction cannot be conducted in traditional logistic models. BNs acted as a tool to represent beliefs and uncertain knowledge about facts and events using probabilities. Through data mining and self‐learning,^22^ BNs can capture probabilistic relations from the “ready‐made” medical records and reveal the potential unknown relationships by separating direct and indirect dependencies.^23^ Before BNs modeling, we initially applied gLASSO‐logistic regression to remove irrelative variables and overcome the issues of local overfitting and node sparsity. So far, LASSO has been widely used for data reduction and model fitting in BNs and other machine learning.^24^, ^25^

The BNs‐based model showed a high accuracy, good interpretability, and strong generalizability. In CSA‐AKI model, the AUCs were beyond to 0.730. While the model performed even better for the prediction of severe AKI with AUCs>0.800 for both internal and external validation. Even compared with the previous AKI model by using other machine learning algorithms, the predictive ability of our model keeps no less striking. *Kate* et al.^26^ compared the AUCs of support vector machines, decision trees, and naive Bayes detection in older patients and found that naive Bayes performed better (AUC: 0.699). *Koyner* et al.^27^ applied a gradient boosting machine model for predicting AKI with the AUC of 0.730 Among general inpatients. Different from these algorithms, our model could infer the probability of AKI occurring based on the acquired prior knowledge even if the known evidence variables were limited. With the accumulation of new data, the structure and parameter of BNs model can keep upgrading continuously. Eventually, we can infer further information or make predictions about unobserved situations and events.

Given the complex and dynamic pathologies of CSA‐AKI, it is challenging to conduct clinical trials. With the causal inference of BNs model, we can simulate the effects of interventions without performing real‐world experiments, which in many cases is expensive and/or impossible. As illustrated in Supplement Figure S3, if medical interventions were taken to avoid excessive ACCT and ultrafiltration volume and correct the postoperative CVP level timely, the incidence of CSA‐AKI should be reduced significantly from 71.6% to 16.1%. Preliminary evidence also suggests that avoidance of hemodynamic instability and careful control of postoperative CVP and mean arterial pressure (MAP) level may help to alleviate the risk of AKI.^28^, ^29^, ^30^ These measures are almost cost‐free and can be implemented as secondary prevention strategies in daily clinical practices.

The hospital‐acquired AKI could have been reduced by a fifth if physicians paid more attention to monitoring electrolytes, identifying patients with high‐risk, and executing kidney prophylaxis.^31^ Leveraging BNs model into the personalized risk prediction can contribute to identifying those at risk for CSA‐AKI (even before SCr rising) and improve patients' postoperative outcomes. Still, the study limitations should be stated. Firstly, participants came from a single medical center. Although we had tried to recruit as many patients as possible, the representativeness are potentially biased. It may contain the further extrapolation of BNs models. Secondly, this study did not collect the medication history of nephrotoxic drugs due to the extensive lack of drug data. The absence of this factor may affect our model's predictive ability to some extent. Thirdly, it has been reported that some novel biomarkers (interleukin 18 and kidney injury molecule‐1) could predict the occurrence of subclinical kidney injury,^32^ which can act as a promising tool to improve the early diagnosis. In future studies, we intend to conduct a multicenter, prospective cohort to collect both clinical and molecular data. The BNs' structure and parameters also will be retrained in the more extensive database with a clear causal time‐sequence.

## 5 CONCLUSIONS

AKI remains a substantially high incidence in patients who underwent cardiac surgeries. We propose a BNs model based on demographic and perioperative risk factors. It not only can reveal the complex relationships between predictors but also infers the individual probability of developing CSA‐AKI. It will facilitate physicians to identify patients with a higher risk of AKI and take protective strategies to improve patients' prognosis.

## CONFLICT OF INTEREST

The authors declare no potential conflict of interests.

## AUTHORS CONTRIBUTIONS

YL participated in the study design, led the data analysis, and drafted the manuscript. JRX participated in the study design and data analysis. LYZ edited the manuscript. WHJ, YMW, and BS were involved in data collection. XQD was responsible for this project and commented on the manuscript.

## Supporting information


**Supplementary Figure S1** Flow chart of the study population selection
**Supplementary Figure S2**. CSA‐AKI and severe AKI candidate variables selection by using gLASSO‐logistic regression
**Supplementary Figure S3**. Bayesian networks inferences under known evidence variables
**Supplement Table S1**. Demographics and clinical features of derivation and validation cohorts
