# Supplementary material for: A novel machine learning algorithm, Bayesian networks model, to predict the high‐risk patients with cardiac surgery‐associated acute kidney injury
Source: Clin Cardiol. 2020 May 12;43(7):752–61. doi: 10.1002/clc.23377 (PMC7368305; doi:10.1002/clc.23377)
Supplement: Supplementary file 1 — Supplementary Figure S1 Flow chart of the study population selection Supplementary Figure S2. CSA‐AKI and severe AKI candidate variables selection by using gLASSO‐logistic regression Supplementary Figure S3. Bayesian networks inferences under known evidence variables Supplement Table S1. Demographics and clinical features of derivation and validation cohorts [file CLC-43-752-s001.docx]

**Supplementary Figure 1. Flow chart of the study population selection**

**4142** patients who underwent cardiac surgery during Jan. 1^st^ 2013 to Dec. 31^st^ 2014

**2361** patients who underwent cardiac surgery during Jan. 1^st^ 2015 to Dec. 31^st^ 2015

**4125** patients over 18 years old

**2348** patients over 18 years old

Excluding **17/13** patients under 18 years old

**3657** patients with surgery and biochemical data

**1926** patients with surgery and biochemical data

**3639** eligible patients

in the **derivation cohort**

**1894** eligible patients

in the **validation cohort**

Excluding **446/408** patients lacking surgery and biochemical test data

Excluding **18/32** patients with less than one SCr test

**4103** patients without heart transplant

**2334** patients without heart transplant

Excluding **22/14** patients with heart transplant

**Supplementary Table 1. Demographics and clinical features of derivation and validation cohorts**

| **Variate** | **Derivation cohort (n=3639)** | | **Validation cohort (n=1894)** | | **χ^2^** | **p-value** | **Standard  difference** |
| --- | --- | --- | --- | --- | --- | --- | --- |
|  | **n** | **%** | **n** | **%** |  |  |  |
| **AKI stage** |  |  |  |  |  |  |  |
| No | 2275 | 62.5 | 1215 | 64.1 | 7.568 | 0.006 | 3.4% |
| Stage 1 | 959 | 26.4 | 536 | 28.3 |  |  | 4.4% |
| Satge 2-3 | 405 | 11.1 | 143 | 7.6 |  |  | 12.3% |
| **Age** |  |  |  |  |  |  |  |
| ≤29 yr | 181 | 5.0 | 88 | 4.6 | 3.352 | 0.060 | 1.5% |
| 30~44 yr | 483 | 13.3 | 226 | 11.9 |  |  | 4.0% |
| 45~59 yr | 1559 | 42.8 | 797 | 42.1 |  |  | 1.5% |
| ≥60 yr | 1416 | 38.9 | 783 | 41.3 |  |  | 5.0% |
| **Gender** |  |  |  |  |  |  |  |
| Male | 2169 | 59.6 | 1089 | 57.5 | 2.284 | 0.131 | 4.3% |
| Female | 1470 | 40.4 | 805 | 42.5 |  |  | 4.3% |
| **BMI** |  |  |  |  |  |  |  |
| ≤18.4 | 200 | 5.5 | 100 | 5.3 | 32.696 | <0.001 | 1.0% |
| 18.5~23.9 | 2161 | 59.4 | 982 | 51.8 |  |  | 15.2% |
| 24.0~27.9 | 988 | 27.2 | 636 | 33.6 |  |  | 14.0% |
| ≥28.0 | 290 | 8.0 | 176 | 9.3 |  |  | 4.7% |
| **Comorbidities** |  |  |  |  |  |  |  |
| Hypertension | 1142 | 31.4 | 668 | 35.3 | 8.550 | 0.003 | 8.3% |
| Diabetes | 366 | 10.1 | 211 | 11.1 | 1.563 | 0.211 | 3.5% |
| **Coronary angiography** |  |  |  |  |  |  |  |
| No | 2004 | 55.1 | 958 | 50.6 | 14.347 | <0.001 | 9.0% |
| Yes (within 3 days) | 408 | 11.2 | 194 | 10.2 |  |  | 3.1% |
| Yes (outside 3 days) | 1227 | 33.7 | 742 | 39.2 |  |  | 11.4% |
| **NYHA grade** |  |  |  |  |  |  |  |
| <2 | 1531 | 42.1 | 427 | 22.5 | 207.735 | <0.001 | 42.7% |
| ≥3 | 2108 | 57.9 | 1467 | 77.5 |  |  | 42.7% |
| **LVEF** |  |  |  |  |  |  |  |
| ≥50% | 3209 | 88.2 | 1619 | 85.5 | 8.186 | 0.004 | 8.0% |
| <50% | 430 | 11.8 | 275 | 14.5 |  |  | 8.0% |
| **Liver function** |  |  |  |  |  |  |  |
| ALT (≥40 U/L) | 484 | 13.3 | 203 | 10.7 | 7.638 | 0.006 | 8.0% |
| AST (≥35 U/L) | 496 | 13.6 | 151 | 8.0 | 38.612 | <0.001 | 18.3% |
| DBiL(≥20.4μmol/L) | 507 | 13.9 | 237 | 12.5 | 2.156 | 0.142 | 4.2% |
| **Renal function** |  |  |  |  |  |  |  |
| SCr (<115 μmol/L) | 3431 | 94.3 | 1787 | 94.4 | 0.010 | 0.919 | 0.3% |
| SCr(≥115 μmol/L) | 208 | 5.7 | 107 | 5.6 |  |  | 0.3% |
| eGFR(≥90 mL/min/1.73m2) | 1722 | 47.3 | 783 | 41.3 | 14.404 | <0.001 | 12.1% |
| eGFR(60~89 mL/min/1.73m2) | 1624 | 44.6 | 943 | 49.8 |  |  | 10.4% |
| eGFR(≤59 mL/min/1.73m2) | 293 | 8.1 | 168 | 8.9 |  |  | 2.9% |
| SUA(≤359 μmol/L) | 1942 | 53.4 | 1057 | 55.8 | 1.861 | 0.172 | 4.9% |
| SUA(360~420 μmol/L) | 790 | 21.7 | 380 | 20.1 |  |  | 4.0% |
| SUA(≥421 μmol/L) | 907 | 24.9 | 457 | 24.1 |  |  | 1.8% |
| Urine Protein | 168 | 4.6 | 105 | 5.5 | 2.283 | 0.131 | 4.2% |
| Urine Erythrocyte | 456 | 12.5 | 236 | 12.5 | 0.006 | 0.966 | 0.2% |
| **Biochemical Test** |  |  |  |  |  |  |  |
| Album (<35 g/L) | 172 | 4.7 | 109 | 5.8 | 2.733 | 0.098 | 4.6% |
| Hemoglobin (<115 g/L) | 400 | 11.0 | 285 | 15.0 | 18.887 | <0.001 | 12.1% |
| Hematocrit (<30%) | 53 | 1.5 | 35 | 1.8 | 1.220 | 0.269 | 3.1% |
| Platelet (≤125*10^9^) | 406 | 11.2 | 197 | 10.4 | 0.732 | 0.392 | 2.4% |
| **Electrolyte disorders** |  |  |  |  |  |  |  |
| Hyponatremia | 62 | 1.7 | 54 | 2.9 | 25.843 | <0.001 | 7.7% |
| Hypernatremia | 124 | 3.4 | 27 | 1.4 |  |  | 12.9% |
| Hypokalemia | 147 | 4.0 | 78 | 4.1 | 3.891 | 0.143 | 0.4% |
| Hyperkalemia | 30 | 0.8 | 7 | 0.4 |  |  | 5.9% |
| **CPB** |  |  |  |  |  |  |  |
| Yes | 2619 | 72.0 | 1521 | 80.3 | 45.949 | <0.001 | 19.7% |
| No | 1020 | 28.0 | 373 | 19.7 |  |  | 19.7% |
| **Surgery type** |  |  |  |  |  |  |  |
| Valve | 1935 | 53.2 | 1118 | 59.0 | 42.016 | <0.001 | 11.8% |
| CABG | 756 | 20.8 | 279 | 14.7 |  |  | 15.9% |
| Aorta | 108 | 3.0 | 68 | 3.6 |  |  | 3.5% |
| Valve + CABG | 167 | 4.6 | 76 | 4.0 |  |  | 2.8% |
| Valve + large vessels | 235 | 6.5 | 154 | 8.1 |  |  | 6.4% |
| Others | 438 | 12.0 | 199 | 10.5 |  |  | 4.8% |
| **ACCT** |  |  |  |  |  |  |  |
| No | 1020 | 28.0 | 373 | 19.7 | 30.690 | <0.001 | 19.7% |
| ≤59min | 1507 | 41.4 | 878 | 46.4 |  |  | 10.0% |
| 60~119min | 1037 | 28.5 | 590 | 31.2 |  |  | 5.8% |
| ≥120 | 75 | 2.1 | 53 | 2.8 |  |  | 4.8% |
| **Ultrafiltration volume** |  |  |  |  |  |  |  |
| ≤1999mL | 1086 | 29.8 | 383 | 20.2 | 76.637 | <0.001 | 22.3% |
| 2000~2999mL | 2214 | 60.8 | 1238 | 65.4 |  |  | 9.4% |
| ≥3000mL | 339 | 9.3 | 273 | 14.4 |  |  | 15.8% |
| **CVP** |  |  |  |  |  |  |  |
| ≤7 mmHg | 1340 | 36.8 | 551 | 29.1 | 24.438 | <0.001 | 16.5% |
| 8~9 mmHg | 867 | 23.8 | 515 | 27.2 |  |  | 7.7% |
| ≥10 mmHg | 1432 | 39.4 | 828 | 43.7 |  |  | 8.9% |

*AKI: acute kidney injury; BMI: body mass index; NYHA: New York heart association; LVEF: left ventricular ejection fractions; ALT: alanine aminotransferase; AST: aspartate aminotransferase; DBil: direct bilirubin; SCr: serum creatinine; eGFR: estimated glomerular filtration rate; SUA: serum uric acid; CPB: cardiac pulmonary bypass; CABG: coronary artery bypass grafting; ACCT: aortic cross-clamp time; APACHE: acute psychology and chronic health status; CVP: central venous pressure.*

**
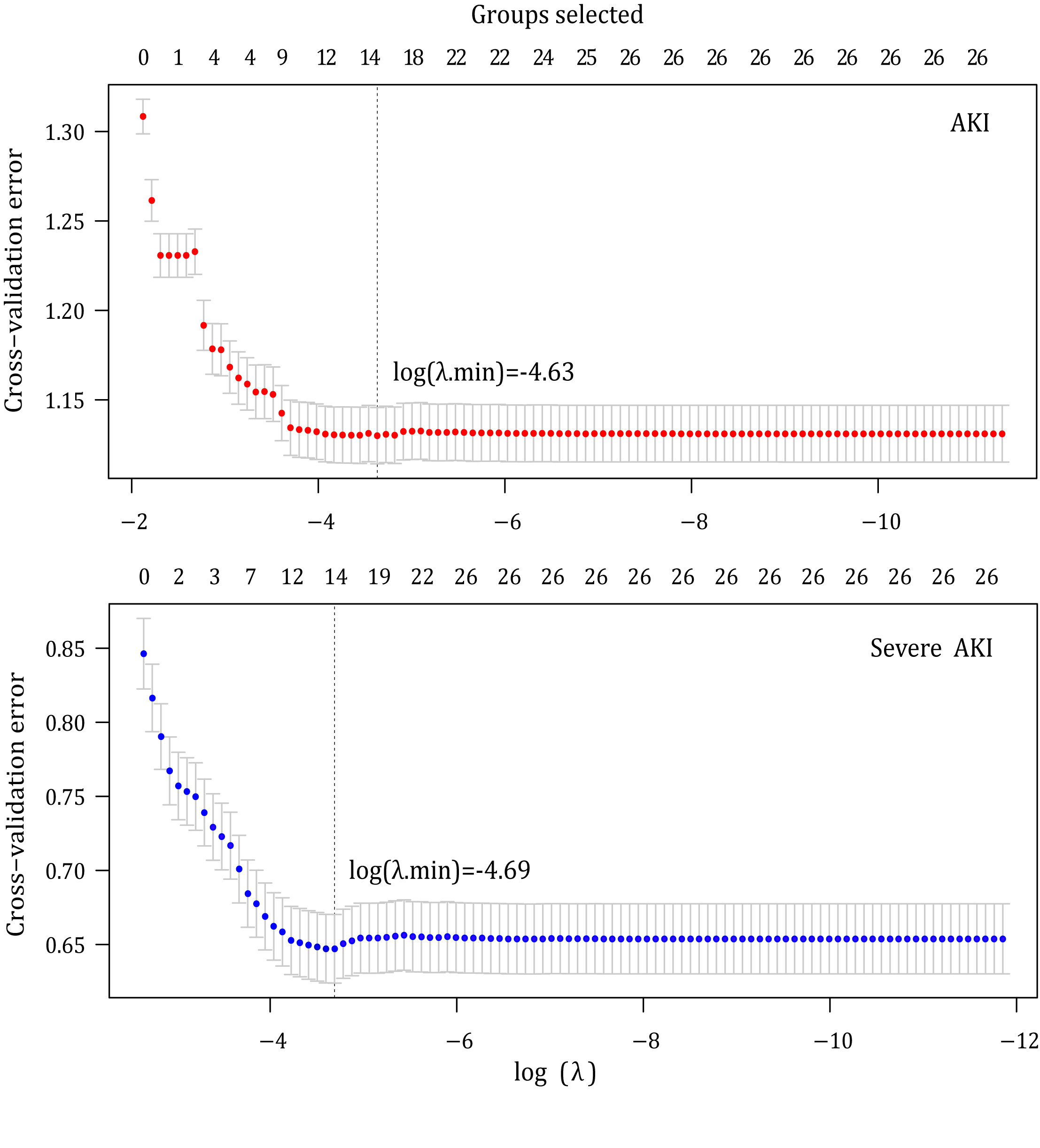
Supplementary Figure 2. CSA-AKI and severe AKI candidate variables selection by using gLASSO-logistic regression**

**
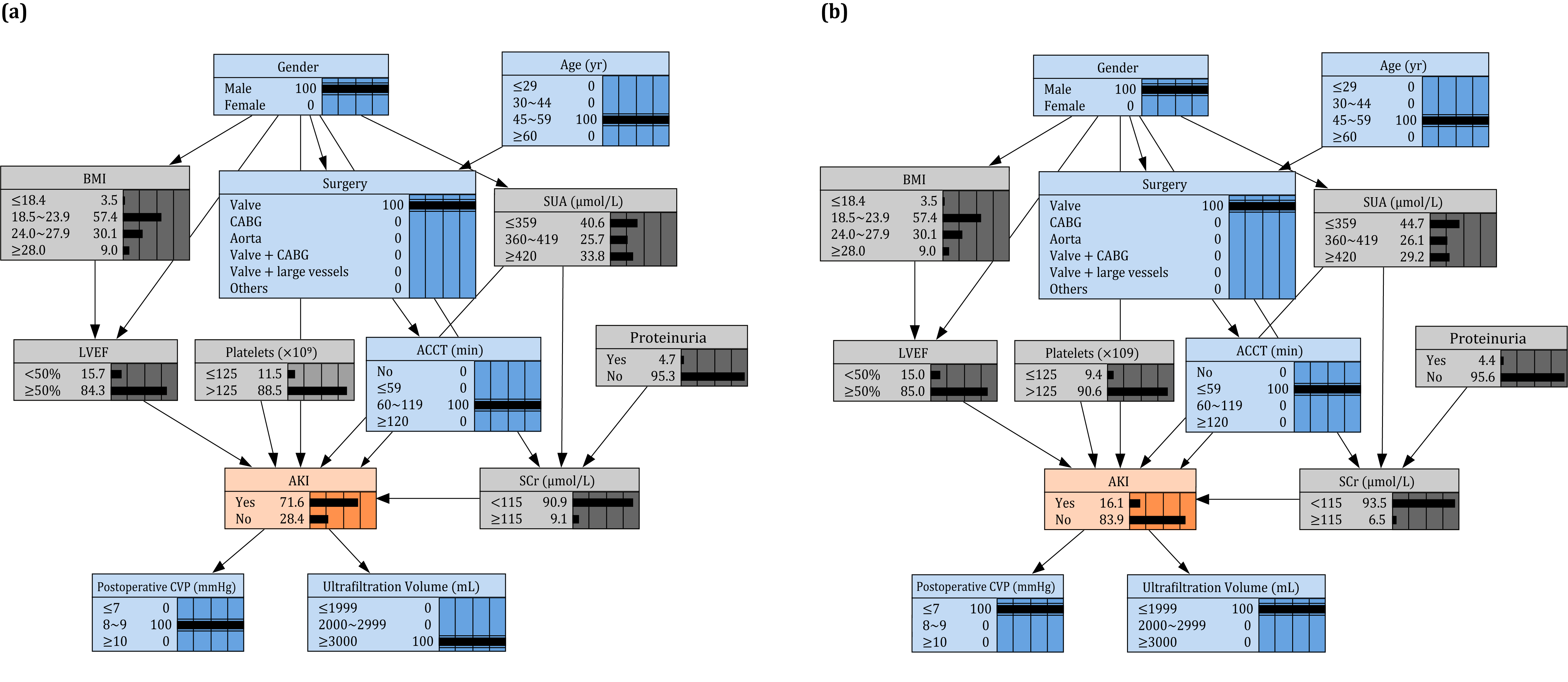
Supplementary Figure 3.** **Bayesian networks inferences under known evidence variables. (**3a: AKI incidence was estimated in given evidence variables of age, gender, surgery category, ACCT, ultrafiltration volume and CVP level. 3b: AKI incidence was estimated if ACCT and ultrafiltration was adjusted in the minimum level and correcting the CVP level timely**)**

*Predictive nodes were colored blue, and the target node was colored orange. AKI: acute kidney injury; BMI: body mass index; LVEF: left ventricular ejection fractions; SCr: serum creatinine; SUA: serum uric acid; CABG: coronary artery bypass grafting; ACCT: aortic cross-clamp time; CVP: central venous pressure.*
